# Comparison of Benchtop and Portable Near-Infrared Instruments to Predict the Type of Microplastic Added to High-Moisture Food Samples

**DOI:** 10.3390/s26010210

**Published:** 2025-12-29

**Authors:** Adam Kolobaric, Shanmugam Alagappan, Jana Čaloudová, Louwrens C. Hoffman, James Chapman, Daniel Cozzolino

**Affiliations:** 1 Centre for Nutrition and Food Sciences (CNAFS), Queensland Alliance for Agriculture and Food Innovation (QAAFI), The University of Queensland, St. Lucia Campus, Brisbane, QLD 4067, Australia; a.kolobaric@uq.edu.au (A.K.); s.alagappan@uq.edu.au (S.A.); louwrens.hoffman@uq.edu.au (L.C.H.); 2 Department of Plant Origin Food Sciences, Faculty of Veterinary Hygiene and Ecology, University of Veterinary Sciences Brno, 612 42 Brno, Czech Republic; h21289@vfu.cz; 3 School of the Environment and Science—Chemistry and Forensic Science, Griffith University, Gold Coast, QLD 4215, Australia; james.chapman@griffith.edu.au

**Keywords:** microplastics, spinach, banana, near-infrared, classification

## Abstract

Near-infrared (NIR) spectroscopy is a rapid, non-destructive analytical tool widely used in the food and agricultural sectors. In this study, two NIR instruments were compared for classifying the addition of microplastics (MPs) to high-moisture-content samples such as vegetables and fruit. Polyethylene (PE), polypropylene (PP), and a mix of polymers (PE + PP) MP were added to mixtures of spinach and banana and scanned using benchtop (Bruker Tango) and portable (MicroNIR) instruments. Both principal component analysis (PCA) and partial least squares (PLS) were used to analyze and interpret the spectra of the samples. Quantitative models were developed to predict the addition of Mix, PP, or PE to spinach and banana samples using PLS regression. The R^2^ _CV_ and the SECV obtained were 0.88 and 0.44 for the benchtop samples, and 0.54 and 0.67 for the portable instruments, respectively. Two wavenumber regions were also evaluated: 11,520–7500 cm^−1^ (short to medium wavelengths), and 7500–4200 cm^−1^ (long wavelengths). The R^2^ _CV_ and the SECV obtained were 0.88 and 0.46, 0.86 and 0.49, respectively, for the prediction of addition in samples analyzed on the benchtop instrument using short and long wavenumbers, respectively. This study provides new insights into the comparison of two instruments for detecting the addition of MPs in high-moisture samples. The results of this study will ensure that NIR can be utilized not only to measure the quality of these samples but also to monitor MPs.

## 1. Introduction

Concerns about food safety and potential contaminants are an important health issue for consumers [1,2]. Recently, consumers are demanding high-quality, nutritious foods and high safety standards for the products they eat [1,2]. Usually, consumers rely on government and authorities to ensure that all food products are not only safe but are also marketed as what they claim to contain [1,2].

Different challenges and catastrophes (e.g., regional wars, pandemics, floods) have influenced food safety, disrupting most of the food supply and value chains. These issues include chemical, biological and personal hygiene, and environmentally related incidents [3,4,5]. Incidents of food products contaminated with industrial pollutants have been well documented and reported not only in the scientific literature but also in the press [3,4,5].

The effects of microplastics (MPs) prevalence on the environment are being evaluated; however, comprehensive monitoring of MPs in feeds, food ingredients, and products, and their effects on human health, remains scarce [3,4,5]. A few studies have reported the presence of MP in sea salt (ranging from 550 to 681 particles/kg), in beer (12–109 fragments/L), honey and sugar (32 fragments/kg), bottled water (28–241 particles/L in returnable plastic bottles) and canned sardines (28.6% plastic polymers in the total particles isolated from canned sardines) [6,7,8,9]. More recently, the main channels of human exposure to MPs have been shown to be linked to the ingestion of food [6,7,8,9,10,11]. This includes the contamination of seafood with MPs [7], as well as commercial processed fish [8], sea salt [9], and honey, beer and food components [6,7,8,9]. Additionally, these food ingredients or products might be contaminated by impurities from processing materials and by contaminants in the packaging, with the second route of exposure being inhalation of air and dust containing MPs [6,7,8,9,10,11].

Plastic contamination has also become a persistent issue across environmental and agricultural systems, arising from the widespread production, use, and disposal of plastic materials [12]. Particularly, post-consumer food waste is a significant contributor to greenhouse gas emissions when sent to landfill [13], yet it also represents an underutilized resource within circular economy frameworks. Such waste streams are typically co-mingled and heterogeneous, originating from households, supermarkets, food precincts, and commercial food processing, which increases the likelihood of plastic contamination [14,15], where these food waste matrices have been reported to range from 0.025% *w*/*w* to 5.3% *w*/*w*, demonstrating the variable nature of post-consumer food wastes.

To ensure the safety of food ingredients and products, it is essential to quantify, monitor, and control MP contamination across the supply and value chains [16]. Large plastic debris and packaging (macroplastics) can be removed through de-packaging processes [16,17]. However, these de-packaging operations across the supply chain can create fragmented materials and generate MPs, defined as plastic particles smaller than 1 mm [16,17]. The presence of MPs in food and organic waste streams is also problematic for downstream valorization pathways such as composting, anaerobic digestion, and the production of bio-based materials, as they can impair process performance, compromise product quality, and lead to environmental dispersal upon land application [18].

In Australia, bananas and leafy greens (e.g., spinach and lettuce) are among the most frequently used and discarded household foods, contributing significantly to household food waste [19]. These materials are rich in water (moisture above 90%) and organic matter, making them ideal representatives of standard food waste matrices. These materials may contain MPs through packaging (for example, plastic wrapping), environmental contamination (such as MPs already present in the environment), process-related contamination during production (for example, wear of machinery), or co-disposal when plastic is knowingly or unknowingly mixed with food waste [19,20].

Near-infrared (NIR) spectroscopy is a rapid, non-destructive analytical tool widely applied in the food and agricultural sectors [21,22]. This technique has been widely used to characterize and measure the chemical composition of different biological samples such as fruit, vegetables, meat, and grains. More recently, it has been used to detect and classify plastic polymers [21,22]. In addition to the measurement of chemical composition, infrared spectroscopy (e.g., NIR, hyperspectral imaging, etc.) has been recognized as an important technique to inform decision makers when utilized as a screening approach. As a screening tool, NIR can provide proximate composition of the sample and helpful information about the safety and authenticity of food ingredients and products, including contamination with MPs [23,24,25,26,27]. This advantage has been explored by researchers and the food manufacturing industry and utilized across the supply and value chains, enabling more samples and analyses to be collected efficiently on suspect samples. Furthermore, one advantage is that these techniques can be easily implemented by the food manufacturing industry [23,24,25,26,27]. The ability of infrared spectroscopy to measure samples in situ provides a qualitative screening tool that enables monitoring and detection of adulterants, defects, or even fraud, as well as contamination with MPs before food ingredients or products enter the food supply and value chains.

Previous studies have demonstrated the potential of NIR spectroscopy to identify the presence or addition of MPs to different types of samples (e.g., soils, environmental samples, etc.). Most scientific papers have focused on adding single polymers to simplified matrices or on scanning samples with a single instrument [28,29]. NIR spectroscopy has been evaluated and demonstrated its potential to assess adulteration and contamination in animal feed [28], in different foods [29], in chicken meat [30], in wheat flour [31], in fish and seafood samples [32], in ash [33], and in corn flour [34]. In most cases, different types of binary mixtures and various concentrations of food ingredients and products were added, and their compositions were predicted using infrared spectroscopy. However, there is still a lack of research examining the influence of MP addition and prevalence on IR spectral analysis and classification results [30,31,32,33,34,35,36].

In this study, two NIR instruments were compared to classify the addition of microplastics (MPs) to spinach and banana. MPs comprising polyethylene (PE), polypropylene (PP), and a mix of both polymers (PE + PP) were added to spinach and banana mixtures and scanned using a benchtop (Bruker Tango) instrument and a portable (MicroNIR) instrument.

## 2. Materials and Methods

### 2.1. Sample Preparation

**Figure 1:**
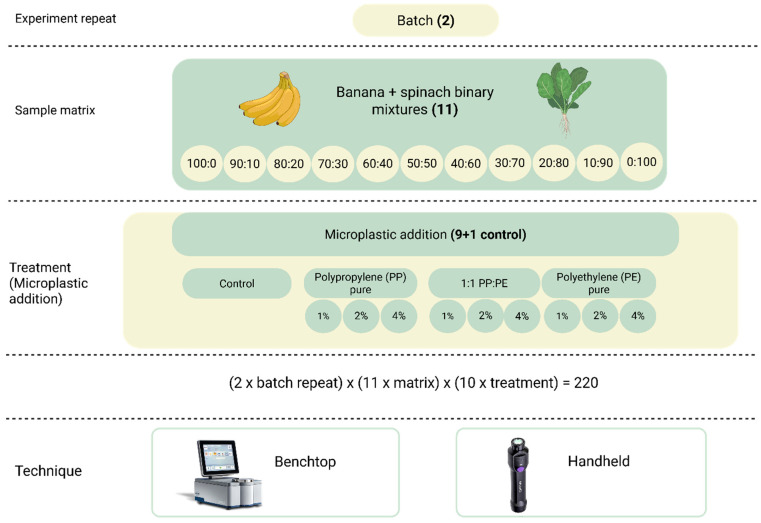
Experimental design, number of samples, and types of instruments used to develop the partial least squares calibration models.

Two separate batches of bananas (*Musa* spp.) and spinach (*Spinacia oleracea*) were purchased from local supermarkets in the Brisbane metropolitan area (Brisbane, QLD, Australia). The binary mixtures used in this study were prepared by homogenizing banana and spinach separately in a coffee grinder (ECH-4gMXA Cuisinart Mini Prep Pro Processor, Stamford, CT, USA) for approximately 10 min, producing a consistent, homogeneous puree for each component. The binary banana–spinach mixtures were then prepared at 11 ratio levels (see Figure 1). Each mixture was weighed gravimetrically to a total mass of 5 g and transferred into 20 mL glass scintillation vials. For example, a 90:10 *w*/*w* banana:spinach mixture was prepared by weighing 4.5 g of banana puree and 0.5 g of spinach puree. After the binary mixtures were prepared, microplastics were introduced to simulate contamination. Polypropylene (PP) and polyethylene (PE), as well as a 1:1 mixture of PE and PP were used. Each polymer type was added at three concentration levels, namely, 1%, 2%, and 4% *w*/*w*, and this was achieved by spiking each of the 5 g banana–spinach mixtures with 0.05 g, 0.10 g, or 0.20 g of MP material, respectively. The mixtures were all homogenized using a vortex mixer for 20 s to ensure even distribution of microplastics within the matrix. Binary mixtures of these materials were used to simulate the chemical compositional variability of real food mixtures.

### 2.2. Grinding and Preparation of Microplastics

Polypropylene (PP) and polyethylene (PE) pellets were obtained from SciPoly (New York, NY, USA). The PP and PE pellets were ground to produce microplastic powders using two different preparation techniques: cryo-milling and cold grinding. For the cold-grinding technique, plastic pellets were frozen at −80 °C and then ground in a Sunbeam Multigrinder II coffee grinder (Sunbeam, Sydney, NSW, Australia) to obtain microplastic powders smaller than 500 µm. Particle size fractions were verified using certified brass sieves with stainless-steel mesh (500 µm aperture, Endecotts, London, UK). For the cryo-milling procedure, PE and PP pellets were milled for 30 s under cryogenic conditions using an IKA A11 basic analytical mill (IKA, Staufen im Breisgau, Baden-Württemberg, Staufen, Germany) laboratory cryo-mill and liquid nitrogen to enhance sample brittleness and obtain finely ground microplastic particles. The cryo-milled and cold-ground PE fractions were then combined in a 1:1 ratio to produce a representative PE microplastic mixture. A mixed polymer microplastic sample (PE + PP) was prepared separately by blending equal masses of the PE and PP microplastic powders (1:1 *w*/*w*).

### 2.3. Near-Infrared Spectra Data Collection

The near-infrared (NIR) spectra were obtained for all samples, including both microplastic-spiked binary banana–spinach mixtures and their unspiked controls, using a benchtop Fourier transform (FT)-NIR spectrophotometer (Tango-R, Bruker Optics GmbH, Ettlingen, Germany) equipped with a gold-coated integrating sphere for diffuse reflectance measurements. Each sample vial was placed directly on the instrument window, ensuring the entire surface was covered by the sample. Spectra were recorded by averaging 64 interferograms at a spectral resolution of 4 cm^−1^ over the range 11,550–3950 cm^−1^, using OPUS software version 8.5 (Bruker Optics GmbH, Ettlingen, Germany). The spectra of the samples were also collected using the same vial in a handheld NIR spectrometer (MicroNIR 1700, Viavi Solutions Inc., Milpitas, CA, USA) operated under controlled laboratory conditions (25 ± 1 °C). The instrument collected spectra in the 950–1600 nm range at 10 nm spectral intervals. Spectral acquisition was managed using MicroNIR Pro v3.1 software (Viavi Solutions Inc.), with an integration time of 50 ms and averaging of 50 scans per spectrum. A Spectralon^®^ reflectance reference was measured every 20 samples to correct for instrument drift and maintain spectral accuracy. Between samples, cuvettes and the sampling window were cleaned with 70% ethanol and dried with Kimwipe™ tissues to avoid cross-contamination.

### 2.4. Data Analysis

**Figure 2:**
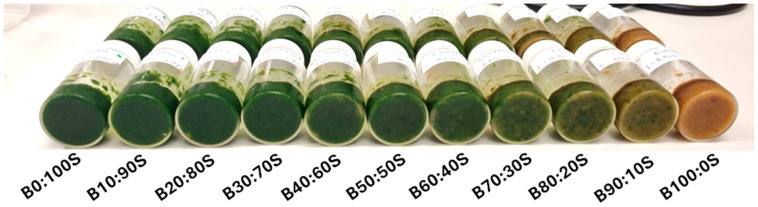
The picture illustrates the series of microplastics added to banana–spinach mixtures.

Spectral data were exported in OPUS and MicroNIR format, and imported into Vektor Direktor (version 2.0, KAX Group, Sydney, NSW, Australia) for pre-processing and chemometric modelling. Before analysis, all spectra were processed using a Savitzky–Golay second derivative (11-point window, second-order polynomial) [36]. Principal component analysis (PCA) was mean-centred and developed using full cross-validation (leave-one-out). Partial least squares regression (PLS) was used to develop models to predict the type of sample (control, mixture, PP and PE). All PLS models were developed using full cross-validation (leave-one-out) [37]. Model performance was evaluated using the coefficient of determination in cross-validation (R^2^_CV_) and the standard error in cross-validation (SECV). The RPD value was calculated and used to assess the performance of the models calculated as the ratio of standard deviation (SD) to the SECV [36,37,38]. A higher RPD value generally indicates better predictive performance (e.g., RPD values greater than 3 are considered adequate for quality control) [36,37,38]. Calibrations were developed using the full NIR spectra from both the benchtop (Tango-R, Bruker Optics GmbH, Ettlingen, Germany) and portable (MicroNIR) instruments, and by selecting two wavenumber regions in the benchtop instrument (Bruker, Tango), defined as the long wavenumber (7500 to 4000 cm^−1^) and short wavenumber (11,500 to 7500 cm^−1^) regions. In this study, a total of 220 samples were prepared and analyzed. This included 22 control samples (all binary banana–spinach mixtures without microplastic addition) and 198 spiked samples (3 levels of microplastic addition × 2 batches × 11 mixture ratios × 3 polymer types). The sample experimental design and an image of the banana–spinach binary mixtures are shown in Figure 1 and Figure 2, respectively.

## 3. Results and Discussion

**Figure 3:**
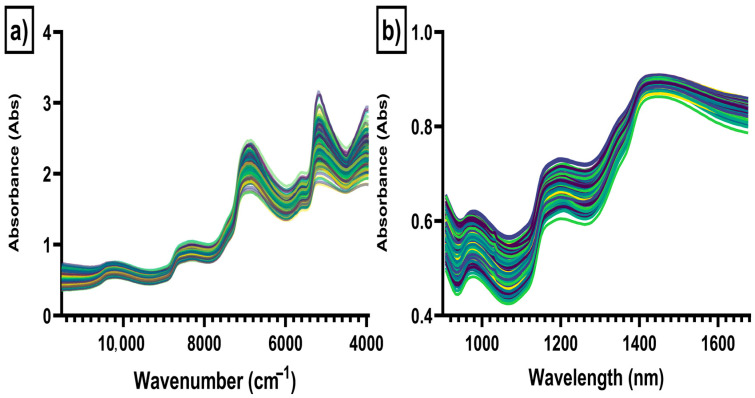
Raw near-infrared reflectance spectra of spinach, banana and microplastic mixtures. Panel (**a**) benchtop (Bruker Tango) instrument; (**b**) portable (MicroNIR) instrument.

Figure 3 shows the raw NIR reflectance spectra of the spinach, banana and microplastic mixture samples analyzed using the benchtop lab instrument (Bruker Tango) (Figure 3a) and the portable (MicroNIR) instrument (Figure 3b). The raw NIR spectra of the samples analyzed in the benchtop instrument showed absorbances at wavenumbers around 10,128 cm^−1^, corresponding to the second overtones of O-H stretching vibrations that can be attributed to water content; at around 8336 cm^−1^, corresponding to C-H methyl groups; at around 6864 cm^−1^, corresponding to N-H aromatic groups, mainly proteins; and at wavelengths around 5568 cm^−1^ associated with O-H and vibration absorption of the C–H aromatic ring. The absorbances around 5168 cm^−1^ are associated with the absorption at 5162 cm^− 1^ and are attributed to the protein molecules’ NH_2_ and CONH_2_ groups [39,40,41,42]. The raw NIR spectra of the samples analyzed with the portable instrument (Figure 3b) showed absorbances at wavelengths around 963 nm associated with O-H overtones (water content); at wavelengths of 1199 nm associated with C-H methyl groups; and at 1428 nm, which is associated with O-H bonds (water content) [39,40,41,42]. In both cases, the raw NIR spectra of the samples analyzed were very similar.

**Figure 4:**
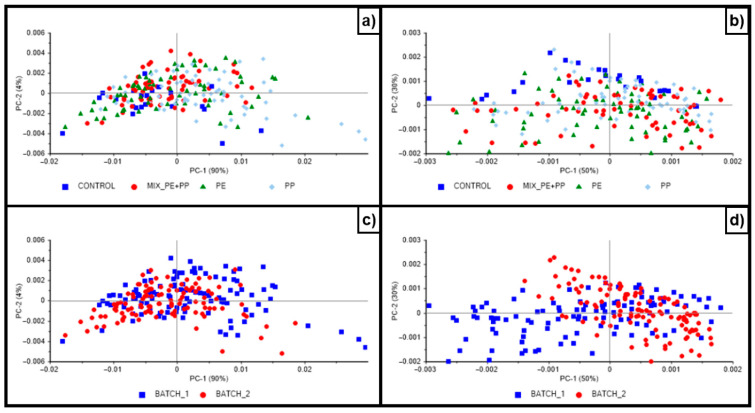
Principal component score plots of the samples analyzed using Bruker Tango benchtop instrument (panels (**a**,**c**)), and MicroNIR portable instrument (panels (**b**,**d**)).

Figure 4a–d show the PCA score plot of the samples. The first three principal components (PC1-PC3) accounted for 96% of the variance in the NIR spectra of the samples analyzed with the benchtop lab instrument. The first three principal components (PC1-PC3) accounted for 85% of the variance in the NIR spectra of the samples analyzed with the portable instrument. No clear separation between samples from the different batches or mixtures was observed using the benchtop instrument. However, a separation between samples from batch 1 and 2 was observed in the set of samples analyzed using the portable instrument. This separation was due to changes in the vial used to collect the spectra during batch 2 (e.g., a change in vial type was detected after completing the experiment). Overall, regarding the separation between samples, similar results were observed by other authors analyzing the addition of MPs to chicken meat samples using NIR spectroscopy [30]. These authors noted that the PCA algorithm may not be an effective method for identifying contamination levels in food samples [30].

**Figure 5:**
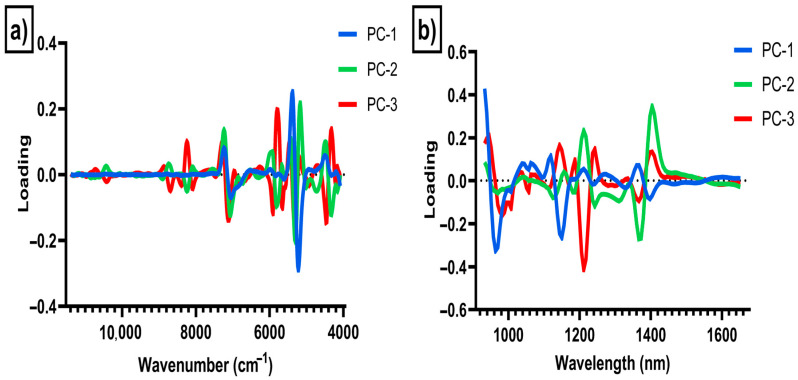
Principal component loadings of the samples analyzed using (**a**) the benchtop (Bruker Tango) instrument, and (**b**) portable (MicroNIR) instrument.

Figure 5a,b show the central wavelengths or wavenumbers (loadings) used to develop the PCA models depending on the instrument used to analyze the samples. The highest loadings in PC1 when the samples were analyzed using the benchtop lab instrument were observed in wavenumbers around 5160 cm^−1^ attributed to NH_2_ and CONH_2_ groups corresponding to protein, and in wavenumbers around 7104 cm^−1^ associated with O-H bands corresponding to water content [39,40,41,42,43]. The highest loadings in PC2 were observed around 8224 cm^−1^ and 5808 cm^−1^ that are associated with the vibration absorption of the C–H aromatic groups, in wavenumbers around 5296 cm^−1^ associated with O-H groups (water), in wavenumbers around 4704 cm^−1^ associated with N-H and C=O combination bands, and around 4320 cm^−1^ corresponding to C-H groups, including lipids, protein and polymers [39,40,41,42,43]. The highest loadings in PC1 for the samples analyzed with the portable instrument were observed at wavelengths around 963 nm associated with O-H groups, at wavelengths around 1149 nm associated with aromatic groups, and at wavelengths around 1397 nm, associated with C-H groups [39,40,41,42,43]. The highest loadings in PC2 were observed at 1124 nm and 1180 nm, which are associated with aromatic and C-H_3_ groups [43], at wavelengths around 1242 nm associated with C-H groups, and at wavelengths around 1372 nm associated with C-H methyl aromatic groups [39,40,41,42,43].

Cross-validation statistics obtained for the quantitative models to predict the addition of PP or PE or a mix of both to spinach and banana were developed using PLS regression are shown in Table 1. The R^2^ _CV_ and the SECV obtained for the prediction of the addition of MPs were 0.88 and 0.44, and 0.54 and 0.67 for the samples analyzed using benchtop and portable instruments, respectively. When PLS calibration models were developed using the portable instrument, samples from batch 1 and batch differences in the cross-validation statistics were observed. The R^2^_CV_ and SECV obtained were 0.84 and 0.40, and 0.64 and 0.48 for batch 1 and batch 2, respectively. These results are not surprising as it was reported that changes in the vial during the analysis can influence the predictive statistics [44]. In addition, the effect of wavelength was evaluated using the data from the Bruker instrument. Two wavenumber regions were evaluated: one from 11,520 to 7500 cm^−1^ (short to medium wavenumbers), and one from 7500 to 4200 cm^−1^ (long wavenumbers). The R^2^ _CV_ and the SECV were 0.88 and 0.46, and 0.86 and 0.49 using the short and long wavenumbers, respectively. The RPD values obtained for the prediction of the addition of MPs to the banana and spinach samples ranged from 2.4 to 3.6. This indicated that the cross-validation models are good enough for predicting the type of MPs added to the samples.

It is well known that the water molecule is a strong absorber in the NIR region, compromising in some cases, the prediction statistics [45,46,47]. When samples contain moisture levels at or above approximately 70 per cent, water begins to fill pore spaces, displacing air [45]. Consequently, solid–air interfaces within air-filled pores normally exhibit large differences in refractive indices, which produce strong scattering at each pore boundary. As water fills the pores, this difference in refractive indices is reduced, determining that light propagates more freely through the material [45]. Because the absorption bands of water increase non-linearly with wavelength, particularly beyond 1400 nm, any increase in optical path length caused by reduced scattering amplifies the apparent absorbance. These combined effects mean that water becomes a dominant spectral contributor, interfering with sample presentation and with measurements above 1400 nm (wavenumber 7143 cm^−1^) [45].

Therefore, when high-moisture samples are analyzed, it is recommended that shorter wavelengths or thicker samples should be used. This can explain the improvements in the PLS models when the short wavelengths were used. Some modern instruments, including diode array or MEMS, make collection of the spectra more reliable [45]. However, it is not clear why poor calibration statistics were obtained (low R^2^_CV_ and high SECV) using the portable (MicroNIR) instrument. These results can be also explained by the different wavelength or wavenumber ranges used, the inherent differences in optical configuration in both instruments, as well as the different types of vials used during the spectra collection.

## 4. Conclusions

Analysis of the samples using both instruments yielded similar results for predicting the addition and type of MP added to the mixtures. However, a change in the vial used to collect the spectra altered the cross-validation statistics for samples scanned with the portable instrument. These results highlight the importance of developing and adhering to standard procedures when calibrating different instruments (benchtop vs. portable) for similar analyses. This study also provides new insights into comparing two instruments for detecting MPs in high-moisture samples. No differences between short and long wavelengths (wavenumbers) in the prediction results were observed. The results of this study will ensure that NIR can be utilized to monitor MPs in such samples. These results can be extended to the detection of MPs in high-moisture samples such as vegetable mixtures or different types of food waste with high moisture content.

## Figures and Tables

**Table 1:** Cross-validation statistics obtained for the quantitative models to predict the addition of PP or PE or a mix of both to spinach and banana developed using partial least squares regression and analyzed using benchtop and portable near-infrared instruments.

	N	R^2^_CV_	SECV	RPD	LV
Benchtop instrument (all samples)	204	0.88	0.44	3.6	7
Portable instrument (all samples)	200	0.67	0.55	2.4	10
Portable instrument (batch 1)	160	0.84	0.40	3.3	11
Portable instrument (batch 2)	110	0.64	0.48	2.8	7
Short to medium wavelengths	200	0.88	0.46	3.0	6
Long wavelengths	199	0.86	0.49	2.8	5

N: number of samples; R^2^ _CV_: coefficient of determination in cross-validation; SECV: standard error in cross-validation; LV: latent variables; RPD: residual predictive deviation (SD/SECV); short to medium wavelengths/wavenumbers: 11,520 to 7500 cm^−1^; long wavelengths/wavenumbers: 7500 to 4200 cm^−1^.

## Data Availability

The raw data supporting the conclusions of this article will be made available by the authors on request.
